# Complete Genome Sequence of Dickeya dadantii subsp. *dieffenbachiae* Strain S3-1, Isolated from a White-Flowered Calla Lily in Taiwan

**DOI:** 10.1128/MRA.00620-21

**Published:** 2021-09-16

**Authors:** Yung-An Lee, Kuan-Pei Chen

**Affiliations:** a Department of Life Science, Fu Jen Catholic University, Xin-zhuang District, New Taipei City, Taiwan, Republic of China; University of Arizona

## Abstract

Erwinia chrysanthemi S3-1 is a bacterial soft rot pathogen of the white-flowered calla lily. The complete genome sequence of the strain was determined and used to reclassify the strain as Dickeya dadantii subsp. *dieffenbachiae*. The sequence will be useful to study plant host-driven speciation in strains of D. dadantii.

## ANNOUNCEMENT

Erwinia chrysanthemi S3-1 is a bacterial pathogen with a higher virulence on white-flowered calla lily (Zantedeschia aethiopica) than other *E. chrysanthemi* strains (1, 2). *E. chrysanthemi* strains have been reclassified into the genus *Dickeya*, which contains 12 species (3–11). In this study, the genome sequence of strain S3-1 was determined and used to identify the strain to the species level of *Dickeya* based on the average nucleotide identity (ANI) (12) and *in silico* DNA-DNA hybridization (isDDH) (13) values.

The genome of strain S3-1 was sequenced using both Illumina MiSeq short-read and Oxford Nanopore Technologies (ONT) long-read sequencing methods. A single colony was cultured overnight at 28°C in Miller’s Luria-Bertani broth medium (Neogen, Lansing, MI), and genomic DNA was extracted using the Genomic-tip 20/G kit (Qiagen, Valencia, CA). The extracted DNA was fragmented and a sequencing library constructed using the Celero EZ DNA-Seq library preparation kit (Tecan Genomics, San Carlos, CA) for Illumina sequencing (MiSeq sequencer; 2 × 300-bp base-paired reads) (Illumina, San Diego, CA). The raw data contained 4,138,542 reads (300 bp each read; *N*_50_, 300 bp) with a total of 1,245.7 Mb. For the long-read sequencing, the short DNA fragments of the same DNA preparation were removed using the short-read eliminator kit (Circulomics, Baltimore, MD), and a sequencing library was prepared using the native barcoding expansion kit 1-12 (EXP-NBD104; ONT) and the ligation sequencing kit (SQK-LSK109; ONT). Sequencing was performed on an ONT MinION device (MIN-101B) with FLO-MIN106 flow cells. In total, 1,663,872 reads (lengths ranging from 2,889 bp to 233 kbp; *N*_50_, 16,136 bp) were generated, comprising 4,807.2 Mb, and trimmed to remove the adapters, low-quality sequences (<Q20), and ambiguous bases using CLC Genomics Workbench v10 (CLC bio, Cambridge, MA). The Nanopore reads were base called, demultiplexed, and adapter trimmed using GridION MinKNOW v21.02.5 software with Guppy v4.3.4 high-accuracy mode. The Illumina trimmed reads and Nanopore reads were used to perform *de novo* assembly using the SPAdes v3.13.0 program (14).

The assembled genome is a 5,065,613-bp circular chromosome (G+C content, 56.5%) and was annotated using the NCBI Prokaryotic Genome Annotation Pipeline v5.2 (15). The chromosome contains 4,337 predicted coding sequences. The ANI and isDDH values were calculated by comparing the genome sequence of strain S3-1 with that of each type species of *Dickeya*, using the ANI Calculator (OAU v1.2) with the OrthoANIu algorithm (16) and a pipeline (http://ggdc.dsmz.de/) for isDDH (13). The species threshold was set to 96% for ANI and 70% for isDDH (12, 13).

The ANI and isDDH values between strain S3-1 and species of *Dickeya* range from 84.64 to 96.74% and 23.4 to 72%, respectively. ANI values above 96% were calculated only with *D. dadantii* strains, and an isDDH value above 70% was calculated only with *D. dadantii* subsp. *dieffenbachiae* NCPPB 2976^T^ (Table 1). Accordingly, strain S3-1 was identified as *D. dadantii* subsp. *dieffenbachiae*. Both strains S3-1 and NCPPB 2976^T^ were isolated from *Araceae* plants. Plant host specificity may exist, and the genome sequence of strain S3-1 will be useful to study host-driven speciation in *D. dadantii* strains.

**TABLE 1:** Average nucleotide identity and *in silico* DNA-DNA hybridization values of strain S3-1 compared with the genome sequences of other *D. dadantii* strains

Strain	GenBank accession no.	Host	ANI (%)	isDDH (%)
*D. dadantii* S3-1	CP076386	Zantedeschia aethiopica (*Araceae*)		
*D. dadantii* subsp. *dieffenbachiae* NCPPB 2976^T^	CM001978.1	Dieffenbachia sp. (*Araceae*)	96.74	72
*D. dadantii* NCPPB 3537	CM001982.1	Solanum tuberosum (*Solanaceae*)	96.46	68.8
*D. dadantii* NCPPB 3937	CP002038.1	Saintpaulia ionantha (*Gesneriaceae*)	96.34	68.4
*D. dadantii* subsp. *dadantii* NCPPB 898^T^	CM001976.1	Pelargonium capitatum (*Geraniaceae*)	96.30	67.4

### Data availability.

The sequence reported here was submitted to the NCBI database under accession no. CP076386, and the raw reads are available in the Sequence Read Archive (SRA) under accession no. SRX11062135 (Illumina MiSeq) and accession no. SRX11062136 (Oxford Nanopore [MinION]), under BioProject accession no. PRJNA734702.
